# Recent Progress in Functional Genomic Research in *Plasmodium falciparum*

**DOI:** 10.2174/138920210791233081

**Published:** 2010-06

**Authors:** Jianbing Mu, Karl B Seydel, Adam Bates, Xin-Zhuan Su

**Affiliations:** 1 Laboratory of Malaria and Vector Research, National Institute of Allergy and Infectious Diseases, National Institutes of Health, Bethesda, MD, USA; 2 Michigan State University, East Lansing, MI, USA

**Keywords:** Malaria, microarray, genome diversity, SNP, recombination, comparative genomics.

## Abstract

With the completion and near completion of many malaria parasite genome-sequencing projects, efforts are now being directed to a better understanding of gene functions and to the discovery of vaccine and drug targets. Inter- and intraspecies comparisons of the parasite genomes will provide invaluable insights into parasite evolution, virulence, drug resistance, and immune invasion. Genome-wide searches for loci under various selection pressures may lead to discovery of genes conferring drug resistance or encoding for protective antigens. In addition, the *Plasmodium falciparum* genome sequence provides the basis for the development of various microarrays to monitor gene expression and to detect nucleotide substitution and deletion/amplification. Genome-wide profiling of the parasite proteome, chromatin modification, and nucleosome position also depend on availability of the parasite genome. In this brief review, we will highlight some recent advances and studies in characterizing gene function and related phenotype in *P. falciparum* that were made possible by the genome sequence, particularly the development of a genome-wide diversity map and various high-throughput genotyping methods for genome-wide association studies (GWAS).

## MALARIA PARASITES AND GENOMES

Malaria is one of the most important tropical parasitic diseases in humans, causing great morbidity and mortality in many developing countries. Approximately 300–500 million clinical cases and ~1 million deaths are reported each year [1]. Human malaria is caused by five species of the *Plasmodium* parasites, namely *Plasmodium falciparum, Plasmodium vivax, Plasmodium ovale, Plasmodium malariae*, and *Plasmodium knowlesi* [2]. Among these, *P. falciparum* causes the most serious forms of the disease. The life cycle of the malaria parasite involves multiple tissues and different stages (sexual and asexual) inside two distinct hosts, mosquitoes and humans. Within its complicated life cycle, the parasite has a diploid genome for a short period of time during its development in the mosquito vector and has a haploid genome throughout the majority of its life cycle. The haploid parasite is amenable to the application of many genetic and genomic tools that play an important role in functional genomics research.

*P. falciparum* has 14 chromosomes containing ~23 million base-pair nucleotides with high AT content (~82%) and is predicted to have approximately 5,500 genes [3-5]. The number of protein-coding genes in *P. falciparum* is comparable to those in free-living yeasts, but the latter organism has a considerably smaller genome in comparison to *P. falciparum*. In addition to differences in coding capacity, the *P. falciparum* genome also has a greater number of hypothetical proteins (~60%) with limited homology to genes with known functions; the functions of these proteins are therefore unknown. Additionally, approximately 1/4 of the current gene models in the *P. falciparum* genome database may contain errors [6,7].

**Fig. (1):**
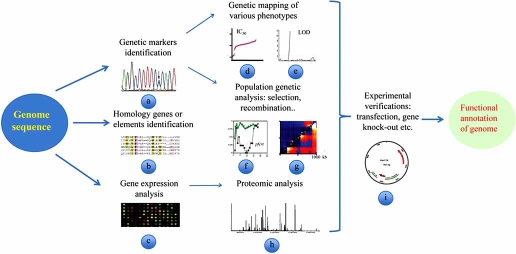
Genetic mapping, comparative genomic analysis, and combination of transcriptomic, epigenomic and proteomic approaches can play important roles in understanding gene functions in *P. falciparum*. **a**. Sequence analysis to identify genetic polymorphisms; **b**. sequence comparison to search for homolog genes or elements; **c**, microarray chips to evaluate the gene expression at mRNA level; **d**, **e**. analysis of different phenotype and genotype data to locate candidate genes/loci associated with drug resistance and other traits; **f**, **g**. population genetic analysis to detect genetic loci under selection or with elevated recombination frequency; **h**. protein expression analysis and association with developmental stages; and **i**. predicated functions of candidate genes can be studied using genetic knock-out and other methods.

Various genomic approaches have been applied to define possible gene functions since the completion of the *P. falciparum* genome sequencing project in 2002 [5] (Fig. **1**), and significant progress has been made. Here we briefly review some of these developments, focusing on progress in genetic mapping using high-throughput genotyping.

## GENOME DIVERSITY AND GENETIC MAPPING

Genetic diversity is considered to contribute to the majority of phenotypic differences; therefore the function of a gene can be inferred either from the linkage or association of genetic polymorphisms to differences in phenotypes [8,9]. Genetic crosses have been successfully applied to identify genes in *P. falciparum *involved in drug resistance, such as *pfcrt* in chloroquine (CQ) resistance [10-12], *pfdhfr* in pyrimethamine resistance [13], and most recently *pfrh5* in determination of the species-specific pathway of *P. falciparum* invasion [14]; however, the cost and intensive lab work of this approach have limited its application for larger-scale functional analysis in human malaria parasites.

With multiple technologic advances, particularly development of high-throughput genotyping since the publication of the *P. falciparum* genome, the genetic markers used for mapping purposes in *P. falciparum* have shifted from the microsatellite (MS) to single nucleotide polymorphism (SNPs). A project of systematic identification of SNP markers in the *P. falciparum* genome was initiated approximately 8 years ago [15,16]. By resequencing approximately 20% of the genome from four parasites (HB3, Dd2, 7G8, and D10) that originated from different geographic locations, ~ 4,000 SNPs were identified after alignment of the sequences with that of 3D7 that was available in the public databases. With the joining of two groups from major sequencing centers at the Broad Institute and the Wellcome Trust Sanger Institute, more parasite genomes have been re-sequenced, and a much larger data set is now available [17,18]. Currently, approximately 180,000 SNPs have been identified from 18 full or partially sequenced *P. falciparum* strains (http://www.ncbi. nlm.nih.gov/projects/SNP/), although some of the SNPs could be errors from sequence alignments that required further verification. Additional strains from global populations are being sequenced using parallel sequencing, which will provide information for better understanding of parasite genome diversity, population structure, and gene functions [19].

In addition to the resequencing approach, high-density tiling arrays have also been developed to study gene expression and genome diversity in malaria parasites [20-23]. Nucleotide change—including nucleotide substitution, deletion, and insertion—results in a reproducible loss or reduction of hybridization signal and therefore allows identification of the region with genetic changes. Several studies have used microarrays to detect nucleotide substitution and copy number variation (CNV) [20,24]. Approximately 20,000 single–feature polymorphisms (SFPs) from 14 field isolates and laboratory lines were identified using an Affymetrix array containing 298,782 oligonucleotide probes [20]. A similar study using a higher-density array (PFSANGER array) containing 2.5 million probes also detected more than 40,000 SFPs from five *P. falciparum* isolates [21]. Moreover, high-throughput SNP typing arrays have been developed for studying parasite population and genetic mapping. Two different platforms are currently available: one utilizes a standard Affymetrix hybridization array [27], and another is based on molecular inversion probe (MIP) technology [25]. Both chips can interrogate ~3000 SNPs and are being upgraded to larger-scale chips that can detect more SNPs. Indeed, a limited numbers of upgraded MIP array chips containing ~8000 SNPs is currently available for public uses at MR4 (http://www.mr4.org/). The Affymetrix standard hybridization chip has been applied to evaluate linkage disequilibrium (LD) and natural selection near the *pfcrt* loci on chromosome 7 in parasite populations from different continents [24], and the MIP array has been used to genotype parasite isolates that have different phenotypic variation. The genotypic data obtained from the MIP array was applied to scan the parasite genome for population recombination events, recent positive selection signatures and association of genetic loci with multiple drug-resistant phenotypes in *P. falciparum* [25]. Undoubtedly, microarrays will play an important role in studying parasite genomes and in genetic mapping in the near future.

Significant insights have been gained in the population structure of malaria parasites as well as mapping candidate genes using currently available SNPs. Population structure or admixture can potentially lead to both false-positive and false-negative results in association studies; thus, structure analysis should be investigated prior to an association study. Analysis of SNPs from chromosome 3 among 99 globally collected parasites showed that malaria parasites could be clustered into different major groups according to their geographic origins independent of time of collection [26]. Similar results were also obtained in a study using the Affymetrix hybridization SNP array, which showed that continental boundaries between parasite populations gave rise to most population structure [27]; however, caution should be exercised in interpreting population structure results when using markers that are likely under selection. For example, SNPs from 49 transporter genes in *P. falciparum,* many of which were likely under drug selection, clustered African parasites into two groups according to parasite response to CQ [26], whereas SNPs in the gene encoding apical membrane antigen 1 (*pfama-1*), a target of host immunity, grouped parasites into six populations that were independent of geographic origin [28]. Therefore, results of population structure analysis will be influenced by the type and nature of genetic marker used.

Recombination can play an important role in shaping the parasite genome. For example, recombination changes the size of the LD region (or haplotype block) in the genome and generates new parasite variants that may evade host immunity. Locating recombination hotspots or coldspots in the genome could therefore provide insight into genome evolution and parasite transmission dynamics. Studies of single chromosome [26] as well as whole genome [25] showed that recombination hotspots were located largely at the ends of the chromosome that contains many multifamily genes such as *var, rifin,* and *stevor*. Many of these recombination hot or cold spots appeared to be conserved among parasite populations, although the population recombination rate varied greatly, ranging from ~400/mb in American parasites to over 10^5^/mb in African parasites. Variation of recombination rate in different populations has been shown to affect the size of LD and haplotype blocks in the genome and therefore should be considered in the design of any association studies [26].

Another important source of information to help define gene function is determining genetic loci that are under recent positive selection. As shown in the human genome, positively selected genes can be classified into groups by broad biologic processes of gene function such as gametogenesis, spermatogenesis, fertilization, metabolism of carbohydrates, lipids, and phosphates, and vitamin transport [29-31]. For malaria parasites, systematic evaluation of selection have been performed at some candidate genetic loci associated with drug response, such as *pfcrt *[32] and *dhfr *[33], as well as at the whole genome level [25]. As expected, genes that confer drug resistance are under strong positive selection, although the strength of selection may vary among different geographic samples. For example, a ~200-kb region containing *pfcrt* was shown to be under positive selection in a population isolated from Africa and Southeast Asia [32], whereas only ~70 kb was under such selection in a Laotian parasite population [33]. Interestingly, several novel genetic loci, including ABC transporters, an iron transporter, a member of SURFIN and some conserved *Plasmodium* proteins, were found under significant positive selection by genome-wide scan [25]. In addition to drug selection, host immunity is also a strong force in shaping the parasite genome, such as generation of polymorphisms in antigenic gene families (diversifying selection). Therefore, screening of highly polymorphic genes in the *P. falciparum* genome may lead to discovery of novel vaccine candidate genes [34], although polymorphism in vaccine candidates may also pose some challenges for vaccine development.

In addition to SNP markers, CNV can also be informative in characterizing gene functions. Copy number changes have been linked to various diseases or biologic processes and can contribute to phenotypic variation in many organisms [35,36]. In *P. falciparum*, recent studies have shown that amplification of *pfmdr1*, *pfgch1*, and *pfdxr *may be important for parasite resistance to mefloquine, antifolate drugs, and fosmidomycin, respectively [20,24,37,38].

To date, genome-wide analyses of genetic diversity in *P. falciparum* has led to identification of several candidate genes or loci for novel vaccine and drug targets. It is expected that many more such loci will be discovered in the near future with the increasing availability of phenotypic and genomic data. In particular, the differences in parasite responses to larger number of chemical compounds can be identified as phenotypes for mapping parasite targets of the chemical compounds and for inferring gene functions [39].

## COMPARATIVE GENOMICS AND HOMOLOGOUS GENE SEARCHES

Along with the dramatic efforts being put forth to search for genomic diversity, comparative analysis of the parasite genome sequences to discover homologous genes plays an important role in elucidating the functions of many predicted proteins. Searches based on biased G/C content and RNA folding potential have led to identification of a large number of noncoding RNA (ncRNA), including splicing RNA, small nucleolar RNA (snoRNA), and telomerase RNA in *P. falciparum* [40,41]. In addition to ncRNA with known functions, several candidate genes appear to be specific to *Plasmodium* spp. and lie adjacent to members of the *var* gene family, possibly contributing to the control of allelic expression of this multigene family. Alternatively, these genes could be acting as recombination hotspots, generating diversity that can contribute to immune evasion. Although no parasite-encoded microRNA (miRNA) genes have been found in *P. falciparum* to date and the function of miRNA-mediated control on gene expression in malaria parasites remains controversial, analysis of potential RNA folding using RNAmicro [42] revealed five novel ncRNA that might act as precursors for miRNA [41]. Further studies are needed to illustrate the role of these ncRNA in gene regulation.

Another example of the use of homology search to characterize gene functions is that of the erythrocyte binding-like (EBL) and reticulocyte-binding-like protein (RBL) gene families that are involved in parasite invasion of erythrocytes. Both of the gene families consist of multiple members located on different chromosomes of *P. falciparum* or other *Plasmodium *spp. Characterization of these families has been greatly enhanced by use of homology searches among different *Plasmodium *spp. A detailed summary of the functional characteristics of these genes can be found in excellent reviews elsewhere [43-45]. More recently, a gene family of intramembrane serine proteases encoded by eight different orthologous genes was discovered in the *P. falciparum* genome [46]. This gene family, termed rhomboid-like proteins (ROMs), is likely involved in host-parasite interaction and is present in the genomes of all Apicomplexan parasites whose genomes have been sequenced. Other families of essential proteases, including those implicated in parasite egress from the erythrocyte such as falcipain-2, plasmepsin II, and a family of putative papain-like proteases termed SERA, have been identified using homology search [47,48]. These proteases may provide key targets for development of new chemotherapeutic treatment strategies.

Comparative genetics have also been important in locating genetic regulatory elements, particularly the apicomplexan AP2 (ApiAP2), in the *P. falciparum* genome [49,50]. These discoveries have drastically changed the landscape of transcriptional regulation in *P. falciparum*. More than 20 ApiAP2 genes have been identified on different chromosomes, all of which were previously annotated as hypothetical proteins [51]. Detailed investigation of two members of this family, PF14_0063 and PFF0200c, suggests an essential role in regulating parasite development [50]. These data, combined with large catalogs of potential *cis*-acting sequences obtained from *in silico* discovery of transcription regulatory elements [52], have enhanced our understanding of the role of transcriptional regulation in *P. falciparum*.

Comprehensive comparative genomics have opened the door for other newly emerging fields in *P. falciparum*, such as secretome and epigenome research. Through close examination of known exported proteins in *P. falciparum*, conserved motifs termed *Plasmodium* export element (PEXEL) and vacuolar transport signal (VTS) have been identified from the parasite genome [53,54]. These elements are necessary for export of hundreds of proteins from the parasites that serve to remodel the host erythrocyte [53-57]. The role of these unique modifications on the infected erythrocyte has already been examined by genetic knockout and functional screens [55]. Molecules with secretion signals might be evaluated for antivirulence targets, as some of these genes are important in knob formation and/or involved in the increased rigidity of the infected erythrocytes [55]. A central portal through which most or all of these exported proteins are transported through the erythrocyte has been recently characterized [58]. Further investigations into the functions of these exported proteins are needed to gain additional insight into the *P. falciparum* secretome.

## VARIATION IN GENE EXPRESSION AT mRNA AND PROTEIN LEVELS

Identification of the genes involved in epigenomic control in the *P. falciparum* genome, such as histone acetylation, methylation, and phosphorylation [59-61], has brought a new level to our understanding of parasite biology and the discovery of new drug targets.

Gene functions can also be predicted by monitoring the dynamic of mRNA or protein expression in combination with related phenotypes or developmental stages. Prior to availability of the whole genome sequence, methods for measuring gene expression level in *P. falciparum* were mostly based on gene-by-gene methods such as northern blot, reverse transcriptase-PCR, and complementary DNA (cDNA) libraries. Although these methods have contributed significantly to our understanding of gene expression and function, DNA microarray—which utilizes the genomic sequence for designing oligonucleotide probes—becomes the favored platform for studying gene expression in malaria parasites due to its higher resolution, reproducibility, and coverage. A microarray transcriptome analysis identified clusters of genes with similar expression patterns that were differentially regulated across the life cycle [62]. Further analysis using an improved clustering approach called ontology-based pattern identification (OPI) in combination with evidence-based annotation revealed 320 gene clusters representing various biologic processes, leading to functional predictions for hundreds previously uncharacterized malaria genes [63-65]. Comprehensive comparison of *in vitro *[52,62,66,67] and *in vivo* [68] gene expression patterns among different parasite isolates as well as expression level polymorphisms (ELPs) in a genetic cross [69,70] have allowed identification of hundreds of transcription regulatory elements and regulatory hotspots. Interestingly, although both *in vitro* and *in vivo *experiments demonstrated that gene transcription in *P. falciparum* parasites is rigidly programmed throughout the erythrocytic cycle, the expression profiles showed dramatic differences for parasites grown in these two different environments [68]. At least three distinct physiologic states, which related to glycolytic growth, starvation response, and a general stress response, were found in *P. falciparum* parasites isolated directly from patients; only one state could match the *in vitro* parasite life stage [68]. A recent study suggested, however, that the "hidden" state of expression might be, in fact, transcripts from gametocytes [71].

The determinants of these transcriptional regulations remain elusive, although increasing identification of genetic regulatory elements and expression quantitative loci (eQTLs) has narrowed down the genetic regions for further investigations. Comparison of the gene expression profile of genetically modified parasites such as drug-selected [72-74] or gene knock-out parasites [75] with their parental wild-type parasites will allow identification of genes that interact with those functionally modified genes.

The mechanism of gene expression variation has been linked not only to DNA sequence alterations but also to epigenetic modifications and other mechanism in *P. falciparum *[76-78]. The most extensively studied gene family is the *var* gene family, which encodes hypervariable surface antigens and displays mutually exclusive expression in infected red blood cells [79,80]. Switching of gene expression states from active to silent or vice versa may be associated with chromatin modifications [77,81], locations of active genes in the nucleus [82,83], and presence of regulatory introns [84,85]. Histone acetylation has been associated with gene activation [82,83], whereas trimethylation of lysine 9 of histone H3 (H3K9me3) was found to silent the genes in *P. falciparum* parasites [81,86]. Genome-wide analysis of histone 3 modification using a chromatin immunoprecipitation (ChIP) assay revealed the cycle-regulated H3K4me3 and H3K9ac at asexual developmental stages in *P. falciparum* [87,88]. Disruption of one of the key genes in chromatin modification (pfSir2) that encodes a histone deactylase caused changes in the H3K9me3 profile, and inhibitors of this enzyme showed high potency against cultured *P. falciparum* parasites *in vitro* [89]. Given the observed differences in the epigenetic code compared with all other organisms studied, *Plasmodium*-specific epigenetic enzyme inhibitors could be explored for new therapeutic agents against *P. falciparum *[89].

Gene functions have also been predicted by large-scale comparative analysis on the protein expression level in *P. falciparum*. Although it can be difficult to obtain sufficient material and to prevent contamination from host cells, two large-scale studies using high-throughput proteomics have detected many stage-specific predicted gene products consistent with results from transcript profiling studies [90,91]. This genomics-based approach has also been widely applied in studies of drug targets [92-94], organelle composition [95], stage- and sex-specific gene functions [23,96,97], validation of data from genomic annotation, post-translational modifications [98,99]. With the completion of its human and insect host genome project, genomic, metabolomic and proteomic analyses of host-pathogen interactions have shed light on many malaria genes’ functions [100-102]. This important research topic has been reviewed elsewhere recently [103-105]. Combined transcriptomic, epigenetic, and proteomic data also allowed uncovering regulatory mechanisms of gene expression in *P. falciparum *[23,99]. With the availability of newer methodologies, analysis of expression variation at the protein level may permit investigation of protein interaction and discovery of targets for new drugs and vaccines.

## FUTURE PROSPECTS

Functional genomic research in *P. falciparum* will undoubtedly continue to contribute greatly to our battle against this deadly parasite. As more phenotypic data become available, the ability to identify gene function will be greatly enhanced by high-throughput, genome-eide approaches. High-throughput assays for parasite phenotypes such as drug response, variation in invasion efficiency, population expression profiling, and variation in parasite metabolites can lead to gene function assignment with the use of genomic data. Moreover, next-generation sequencing methods are emerging as the dominant genomic technologies and can be applied in a variety of contexts for functional genomics research, including whole-genome sequencing, targeted resequencing, deep transcriptome analysis to complement microarray analysis, and other genome-wide approaches. In addition, application of novel genetic manipulation tools such as transposon mutagenesis (piggyBac) [106] and improved transfection methods [107] will be extremely valuable for generating functional mutations and for verifying gene functions.

## ABBREVIATIONS

cDNA: = Complementary DNA
ChIP: = Chromatin immunoprecipitation
CNV: = Copy number variation
CQ: = Chloroquine
EBL: = Erythrocyte binding-like
ELP: = Expression level polymorphisms
LD: = Linkage disequilibrium
MIP: = Molecular inversion probe
miRNA: = microRNA
MS: = Microsatellite
ncRNA: = Noncoding RNA
OPI: = Ontology-based pattern identification
PEXEL: = *Plasmodium* export element
RBL: = Reticulocyte-binding like protein
SFP: = Single-feature polymorphism
snoRNA: = Small nucleolar RNA
SNP: = Single nucleotide polymorphism
SP: = Sulfadoxine-pyrimethamine
VTS: = Vacuolar transport signal
